# Nature: A Substantial Source of Auspicious Substances with Acetylcholinesterase Inhibitory Action

**DOI:** 10.2174/1570159X11311040003

**Published:** 2013-07

**Authors:** Ilkay Erdogan Orhan

**Affiliations:** 1 Department of Pharmacognosy, Faculty of Pharmacy, Gazi University, 06330 Ankara, Turkey;; 2 Department of Pharmacognosy and Pharmaceutical Botany, Faculty of Pharmacy, Eastern Mediterranean University, Gazimagosa, The Northern Cyprus via Turkey

**Keywords:** Acetylcholinesterase, cholinesterase inhibition, neuroprotection, herbal, animal, microorganism

## Abstract

Acetylcholinesterase (AChE) (EC 3.1.1.7) is an important enzyme that breaks down of acetylcholine in synaptic cleft in neuronal junctions. Inhibition of AChE is associated with treatment of several diseases such as Alzheimer’s disease (AD), myasthenia gravis, and glaucoma as well as the mechanisms of insecticide and anthelmintic drugs. Several AChE inhibitors are available in clinical use currently for the treatment of AD; however, none of them has ability, yet, to seize progress of the disease. Consequently, an extensive research has been going on finding new AChE inhibitors. In this sense, natural inhibitors have gained great attention due to their encouraging effects toward AChE. In this review, promising candidate molecules with marked AChE inhibition from both plant and animal sources will be underlined.

## INTRODUCTION

Acetylcholinesterase (AChE) (EC 3.1.1.7), also known as acetylhydrolase, is a serine protease type of enzyme that catalyzes breaking down of acetylcholine (ACh) into choline and acetic acid and terminates neurotransmission at cholinergic synapses. The enzyme is known to have a fast catalytic activity with an ability to hydrolyze 25.000 ACh molecules *per* second [1]. Elucidation of the three-dimensional structure of **AChE **isolated from *Torpedo californica* (electric eel) (*Tc*AChE) in 1991 by Sussman *et al*. accelerated the research on this enzyme, which further led to a series of theoretical and experimental studies [2]. AChE-coding sequences have been cloned up to now from a number of simple to complex organisms including insects, nematodes, fish, reptiles, birds, and several mammals including human being [3]. In fact, the crystal structures of AChE from various sources such as mouse, *Drosophila*, electric eel, and human being have been demonstrated to possess similar homology to each other at high rate [4-7]. The enzyme is present in the cholinergic junctions localized at central nervous system as well as peripheral sites such as erythrocytes, and muscles. The active site of AChE, also called the esteratic subsite lined by 14 conserved amino acid residues, consists of two separate ligand binding subsites: an acylation site at the bottom of the gorge where substrate is hydrolyzed and a peripheral anionic site (PAS) located at mouth of the gorge, both of which are critical to the breakdown of ACh [8,9]. Inhibitors of AChE tend to bind either to the active site or to the PAS, an allosteric site containing the indole of Trp279 as the principal component that serves to bind a molecule of ACh to the enzyme [10].

AChE possesses multifunctional properties. Needless to say, inhibition of AChE is the most popular as the treatment strategy against Alzheimer’s disease (AD), which is characterized by reduction in the activity of the cholinergic neurons according to cholinergic hypothesis [11,12]. Actually, AChE inhibitors constitute the only drug class approved by the U.S. Food and Drug Administration (FDA) for the treatment of AD at the moment. In addition to wide-range use of AChE inhibitors towards AD, certain chemical classes of pesticides, such as organophosphates and carbamates, work by interfering or inhibiting irreversibly this enyzme, which is expressed in all invertebrate and vertebrate animals as a key enzyme of the cholinergic system [13,14]. Another treatment approach for utilization of AChE inhibitors has been stated against myasthenia gravis (MG) through inhibition of peripheral AChE [15,16], while AChE inhibitors are also considered as the third line drugs for the treatment of glaucoma, which have been described to be better agents with fewer local and systemic adverse effects [17].

Due to its unique biochemical properties and physiological significance, AChE is an attractive topic of intensive investigations throughout the world. The research on discovering new inhibitors of AChE has been focused on the compounds of both synthetic and natural origins. In this review, a special focus will be reflected on promising compounds with AChE inhibitory effect from natural sources including plants, animals, and microorganisms along with a brief emphasize about the conventional AChE-inhibiting natural compounds already in use.

### AChE Inhibitors of Plant Origin

The first example of a clinically-used natural compound inhibiting AChE was physostigmine (eserine), an alkaloid isolated from the seeds of *Physostigma venenosa* Balf. (Fabaceae), known as Calabar bean, to treat AD, MG, glaucoma, and delayed gastric emptying. Nonetheless, its short half-life, narrow therapeutic index as well as side effects such as nausea, vomiting, headaches, diarrhea, and dizziness led to its withdrawal from clinical use for this purpose [16,18,19]. Following physostigmine, cholinesterase inhibitors became the first line drugs used to treat the symptoms of AD in early 1990s and galanthamine, isolated from the bulbs of *Galanthus woronowii* Los. (Amaryllidaceae) (snowdrop), was the second plant-originated alkaloid with AChE inhibitory effect that has found a widespread clinical application after the synthetic drugs tacrine, rivastigmine, and donepezil [19-21]. Later on, huperzine A (HupA), the alkaloid type of AChE-inhibiting compound isolated in 1986 from the Chinese herb *Huperzia serrata* (Thunb. Ex Murray) Trev. (Lycopodiaceae/Huperziaceae), has been the most recent anti-AChE compound with highly promising clinical use in near future [22]. It is a reversible, potent, and selective inhibitor of this enzyme with a better therapeutic index than physostigmine and tacrine [23]. In fact, HupA (shuangyiping) is already in clinical use for symptomatic treatment of AD in China in a tablet form developed in 1996 and approved as the new drug by the State Administration of Traditional Chinese Medicine [24]. More details can be found on cholinesterase inhibitory activity of the aforementioned compounds in many excellent reviews that have been published up to date [12, 20, 25-28]. In addition to the known ones, the extensive research on plants have been still going on finding new inhibitors from the plant kingdom. Many herbal compounds from different chemical classes were identified with remarkable AChE inhibitory potentials as some examples can be found below:

### Withanolides

**Fig. (1):**
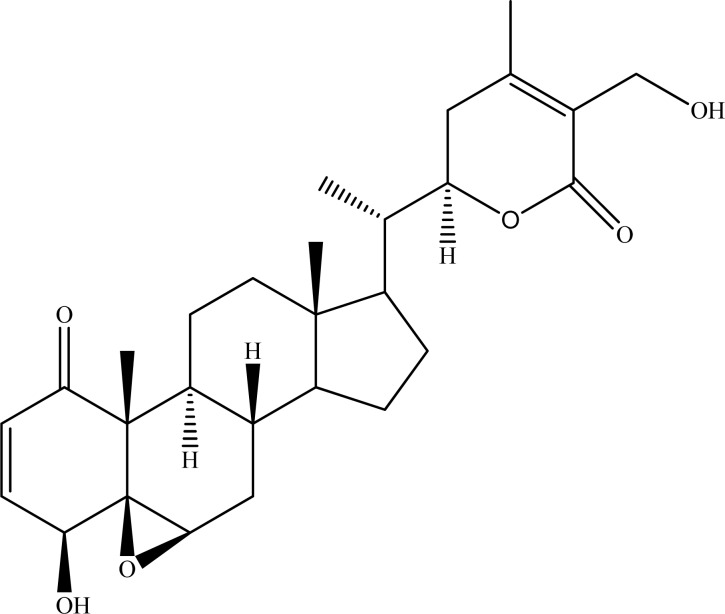
Withaferin A.

*Withania somnifera* Dunal. (ashwaganda or Indian ginseng) (Solanaceae) is a popular plant used for improvement of memory and cognitive enhancement in Indian traditional medicine [29]. Withanolides, firstly isolated from *W. somnifera*, are the naturally-occurring steroid lactones having ergostane skeleton. Several studies have underlined marked AChE inhibitory activity of the withanolide derivatives. For instance; two new 6a,7a-epoxy-3b,5a,20b-trihydroxy-1-oxowitha-24-enolide **(1)** and 5b,6b-epoxy-4b,17a,27-trihydroxy-1-oxowitha-2,24-dienolide **(2)** and four known withanolides withaferin A **(3)**, 2,3-dihydrowithaferin-A **(4)**, 6a,7a-epoxy-5a,20b-dihydroxy-1-oxowitha-2,24-dienolide **(5)**, and 5b,6b-epoxy-4b-hydroxy-1-oxowitha-2,14,24-trienolide **(6) **isolated from the methanol extract of *W. somnifera *collected in Karachi (Pakistan) were tested for their possible AChE inhibitory effect and only four of the compounds, whose IC_50_ values varied between 50±2.0 and 161.5±1.1 µM, were found to display moderate level of inhibition towards AChE as compared to galanthamine (IC_50_ = 0.50±0.001 µM) employed as the reference [30]. Among them, withaferin A **(3)** (Fig. **1**), a known metabolite, was the most active in this assay (IC_50_ = 50±2.0 µM). Previously, administration of withaferin A in combination with several sitoindoside derivatives caused an augmentation in AChE activity in the lateral septum and globus pallidus in mice, whereas this combination reduced AChE activity in the vertical diagonal band [31]. 

**Fig. (2):**
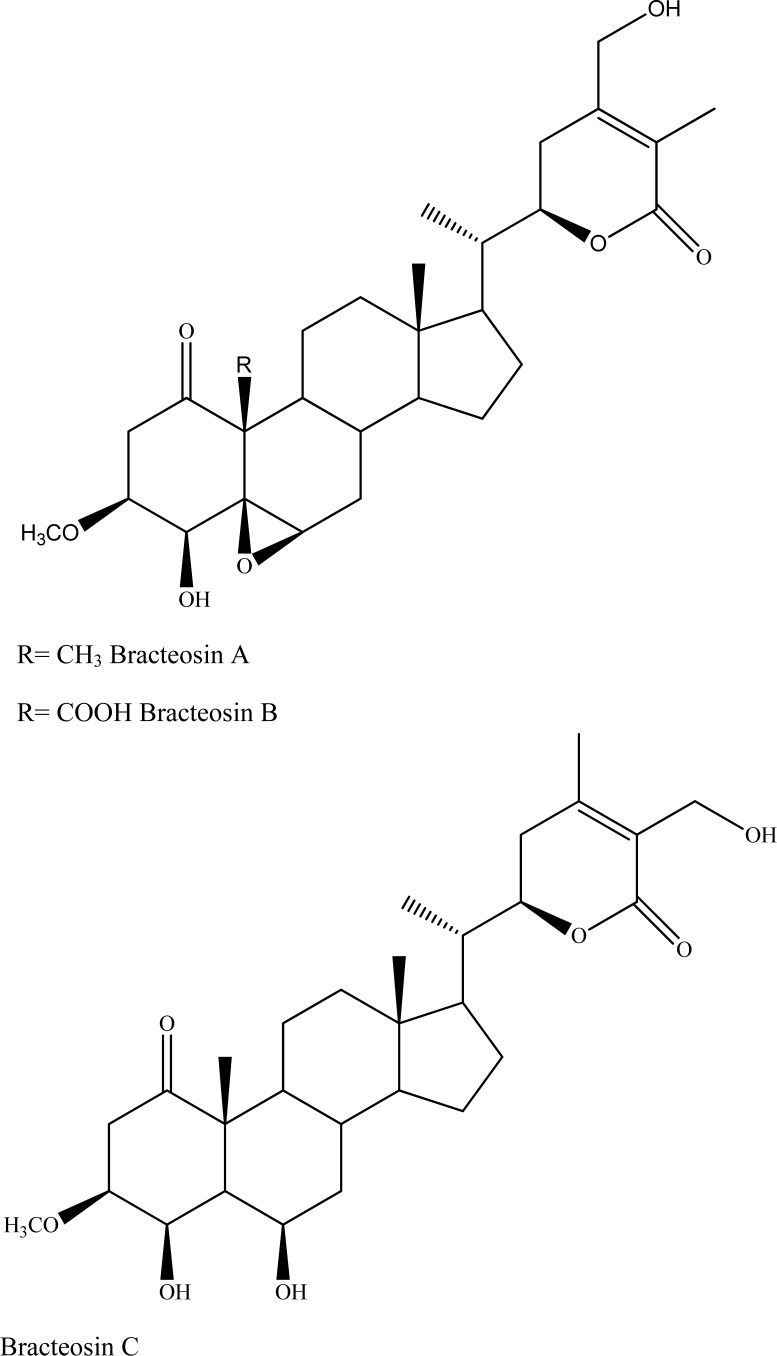
Bracteosins A-C.

By the same research group, three new withanolide derivatives (bracteosins A-C) were isolated from *Ajuga bracteosa* Wall ex Benth. (syn. *A. remota*) (Lamiaceae) of Pakistani origin and subjected to *in vitro *AChE inhibition assay [32]. The results revealed that bracteosins A-C (IC_50_ = 25.2±0.4, 35.2±0.5, and 49.2±0.8 µM, respectively) displayed a siginificant inhibitory action in a concentration-dependent manner (Fig. **2**). 

In a related study by these researchers on the withanolide derivatives **(1-6)** isolated in their previous work [31], the active derivatives were further investigated against AChE by *in silico* methods using molecular docking simulations [33]. Molecular docking study findings indicated that all the ligands (the active compounds) were entirely embedded inside the aromatic gorge of AChE, whilst compounds **1**, **3**, and **5** extended up to the catalytic triad. Besides, Lineweaver-Burk and Dixon plots and their secondary replots revealed that compounds **1**, **3**, and **5** are the linear mixed-type inhibitors of AChE. Hence, these studies led to a statement that withanolides appear to be a new class of AChE inhibitors. 

In a study published in 2007, Vinutha *et al*. reported that the root methanol and water extracts of *W. somnifera* from India exhibited 75.95±0.16% and 24.60±0.38% of inhibition against AChE, respectively, at concentration of 100 µM. The authors concluded that the active principles inhibiting AChE could be withanolides [34]. In a recent study, the crude methanol extract of the whole plant of *W. somnifera* collected from Khyber tribal area (Pakistan) together with its hexane, chloroform, ethyl acetate, butanol, and water fractions were tested *in vitro* against AChE and the crude methanol extract was found to have IC_50 _value at 76±0.16 µg/mL, while IC_50 _values of the chloroform, ethyl acetate, butanol, and water fractions were calculated as 69±0.20, 78±0.20, 97±0.23, and 111±0.14 µg/mL, respectively. The hexane fraction was inactive, whereas the most active one against AChE was the chloroform extract in comparison to the reference drug, galanthamine (IC_50_= 22.6±0.09 µg/mL) [35]. In a similar work [36], the aqueous extract of *W. somnifera* roots from India inhibited AChE in concentration-dependent fashion with maximum inhibition (86.7%) detected at the final assay concentration of 1 mg/mL. The kinetic studies revealed that the extract caused a mixed non-competitive inhibition towards the enzyme. 

**Fig. (3):**
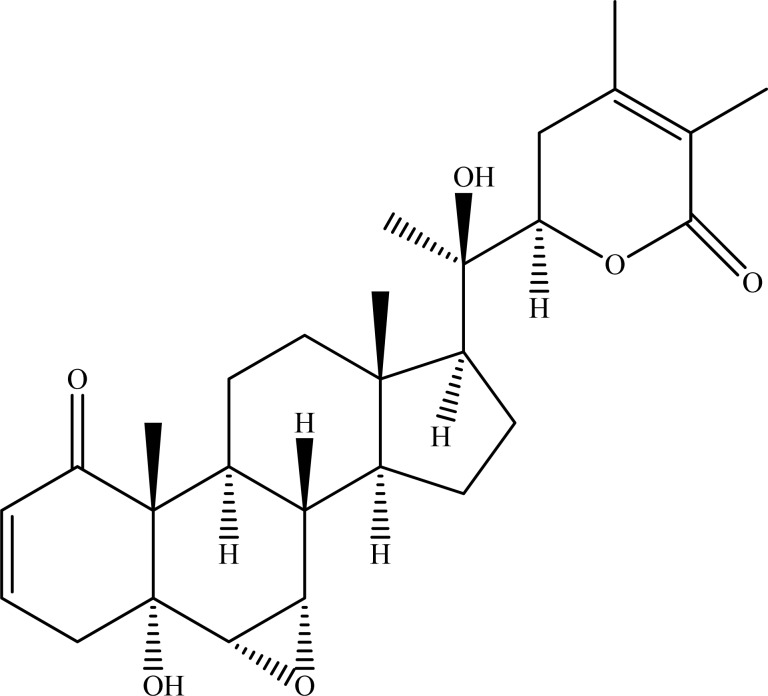
Withanolide A.

On the other hand, withanolide A (Fig. **3**) showing strong AChE inhibition was applied to molecular docking studies and high-binding affinity of the ligand (withanolide A) to receptor was observed. Furthermore, in this computational study, long *de novo* simulations exerted interaction of the ligand with the residues Thr78, Trp81, Ser120, and His442 of human AChE [37]. 

### Curcuminoids

**Fig. (4):**
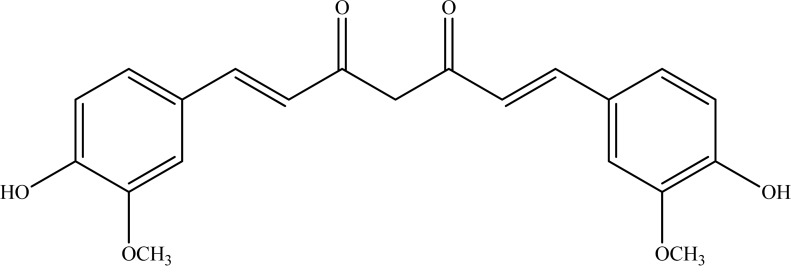
Curcumin.

The rhizomes of *Curcuma longa* L. (Zingiberaceae), known as turmeric, is indigenous to the Southeast Asian countries and used as culinary spice. The principal chemical ingredients in turmeric have been identified as curcuminoids consisting of curcumin (diferuloyl methane) (Fig. **4**), bisdemethoxycurcumin (5%), and demethoxycurcumin (15%) and the dominant component in this mixture is curcumin (75-85%) [38]. 

Curcumin has received a considerable attention from the scientists due to its strong antioxidant, anti-inflammatory, metal-chelator, anticancer, and other biological effects [39-43]. In addition, curcuminoids have been broadly studied for their neuroprotective properties using different experimental methods [44-48]. AChE inhibitory effect of curcuminoids were evaluated by Ahmed and Gilani and IC_50 _values of the curcuminoids were reported as 67.69 µM for curcumin, 16.84 µM for bisdemethoxycurcumin, and 33.14 µM for demethoxycurcumin [49]. The IC_50 _value was found as 19.67 µM for curcuminoids, which is the mixture of these three individual compounds. Since bisdemethoxycurcumin appeared to be more effective than the parent curcuminoids, the authors commented that the additional methoxy group (-OCH_3_) on the structure of bisdemethoxycurcumin may influence the level of AChE inhibitory activity. According to the literature survey, this was the only report on *in vitro* AChE inhibitory action of curcuminoids up to date. However, it is interesting to note that the ethanol extract of the rhizomes of *C. longa* of Pakistani origin was found to be totally inactive against AChE in an earlier study [50]. In another report, curcumin was tested against arsenic-induced neurotoxicity in rats, which causes cognitive deficits [51]. According to the results, curcumin reduced AChE activity from 46% to 41% in arsenic-treated rats. It was stated that curcumin significantly modulates arsenic-induced cholinergic dysfunctions in brain that may help to explain neuro-protective effect of this compound. In a similar work [52], curcumin was assessed in rats for its effects on aluminum-induced alterations in ageing-related parameters including AChE activity. The evidence showed that co-administration of curcumin and aluminum lowered the levels of lipid peroxidation, activities of protein kinase C and AChE. 

### Tanshinones

**Fig. (5):**
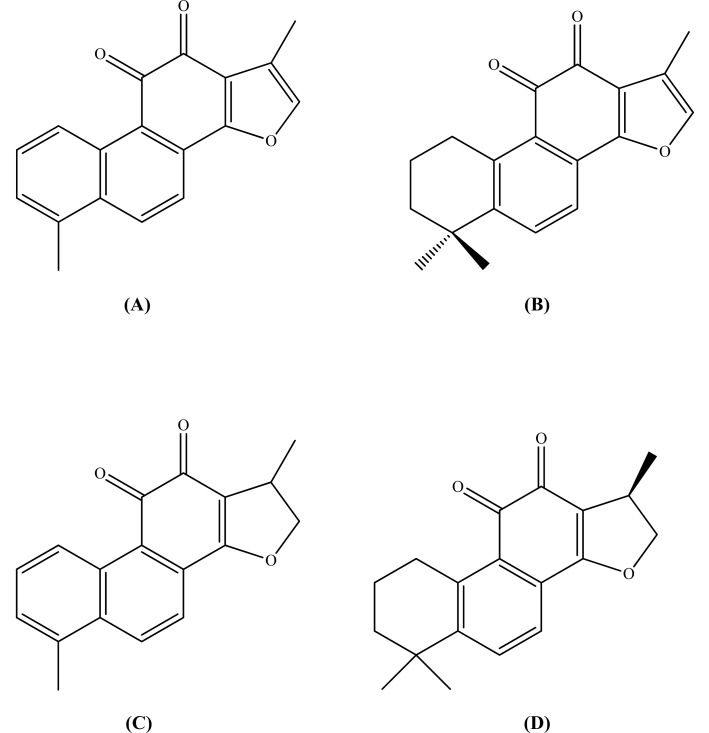
Tanshinones; tanshinone I (A), Tanshinone IIA (B), dihydrotanshinone I (C), and cryptotanshinone (D).

The roots of *Salvia miltiorrhiza* Bunge (Lamiaceae) are locally known as ‘Danshen’ in the traditional Chinese herbal medicine. Tanshinones, a mixture of diterpenoids including tanshinone I, dihydrotanshinone I, tanshinone IIA, and cryptotanshinone, are the major active ingredients of *S. miltiorrhiza* extract (Fig. **5**) [53, 54]. 

A wide array of the reports pointed out to neuroprotective effect of *S. miltiorrhiza* (or danshen) as well as its individual components [55-59]. The acetone extract of danshen was tested in AChE inhibitory activity assay and activity-guided fractionation of the extract led to isolation of tanshinones (tanshinone I, dihydrotanshinone I, tanshinone IIA, and cryptotanshinone) with remarkable AChE inhibitory effects [60]. Among them, dihydrotanshinone I and cryptotanshinone displayed concentration-dependent inhibitory activities with IC_50_ values of 1.0 and 7.0 µM, respectively. Besides, in the same report, clogP values of tanshinones pointed out to potential of blood-brain barrier passing ability of these compounds. In a later study by Wong *et al*. [61], cryptotanshinone was also shown to reversibly inhibit AChE of human origin with IC_50_ value of 4.09 µM. In continuation with this report, the same research group investigated inhibitory characteristics of cryptotanshinone and dihydrotanshinone towards human AChE using molecular docking models and kinetic analysis, which led to the evidence that both of the compounds exerted a mixed non-competitive inhibition for human AChE, while they had mainly hydrophobic interactions with the allosteric site inside the active-site gorge of the enzyme [62]. The danshen ethanol extract and tanshinones were examined in another work for their AChE inhibitory effects using rat brain homo-genates as an enzyme resource [58]. The researchers concluded that the extract, total tanshinones as well as tanshinone I and dihydrotanshinone I exhibited remarkable *in vitro *inhibition on the enzyme, which was in accordance with the relevant previous reports. On the other hand, cryptotanshinone was again found to inhibit AChE activity for 3 h and 15, 16-dihydrotanshinone I for 6 h in *ex vivo* conditions [63].

### Quercetin

**Fig. (6):**
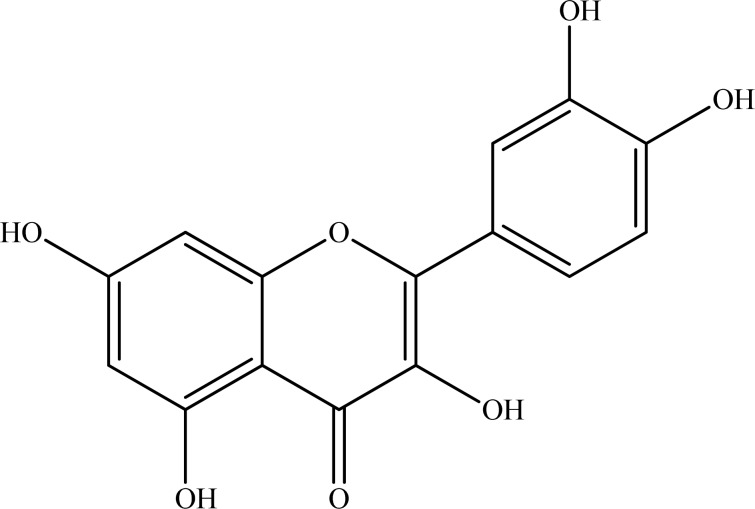
Quercetin.

Quercetin (Fig. **6**), whose name was derived after its isolation from *Quercus* sp. (oak), is a flavonoid derivative widely found in plants and ubiquitously present in many foodstuffs such as mainly colored fruits, vegetables, wine, etc. It belongs to flavonol subtype of flavonoid family and also named as “bioflavonoid” due to its broad spectrum health effects [64-69]. 

Moreover, quercetin has been reported to possess neuroprotective effects approved by different mechanisms and has recently become a more striking target for neurobiological research [70-75]. In our former study [76], we identified quercetin with a moderate *in vitro* inhibition against AChE (76.2±0.99%) at final concentration of 100 µg/mL and, then, we further investigated AChE inhibitory effect of four flavonoid and xanthone derivatives including quercetin and identified once more inhibition of AChE by quercetin in concentration-dependent fashion (IC_50_=353.86 µM) [77]. Our findings on enzyme kinetics also revealed that quercetin had a selective inhibition towards the enzyme in competitive manner. The molecular docking simulations indicated that the main phenylchromen part of quercetin binds with several hydrogen bonds to the important amino acid residues of PAS of AChE, while it had hydrophobic interactions with Ser125, His447, and Glu202, which are some of the prominent residues of PAS of AChE. 

On the other hand, quercetin has been reported to be active principal of a number of extracts having AChE-inhibiting features. For instance; in a screening of 180 extracts obtained from the medicinal plants from Korea for their AChE inhibitory activity, the ethyl acetate extract of whole plant of *Agrimonia pilosa* Ledeb. (Rosaceae) yielded tiliroside, 3-methoxy quercetin, quercitrin, and quercetin, among which quercetin in particular showed twice more inhibition as compared to dehydroevodiamine (the reference compound in this assay) [78]. Among the components isolated from *Polygonum sachalinensis* F. Schmidt ex Maxim (Polygonaceae) collected in Switzerland, quercetin-3-O-β-D-galactopyranoside was revealed to inhibit the enzyme to some extent (55.6%), whereas quercetin-3-O-arabinopyranoside was inactive [79]. In fact, the activity of quercetin-3-O-β-D-galactopyranoside (IC_50_ = 0.068 mM) was not significantly different from that of its aglycone quercetin (IC_50_ = 0.060 mM). However, considering IC_50_ values of quercetin and quercetin-3-O-β-D-galactopyranoside, neither of them was comparable with galanthamine (IC_50_ = 0.0009 mM) employed as the reference, which was consistent with our previous study [77]. In a similar study [80], quercetin in addition to four other flavonoids isolated from the buds of *Cleistocalyx operculatus* (Roxb.) Merr. et Perry (Myrtaceae) was tested against AChE and found to inhibit this enzyme with IC_50_ value of 25.9 µM. A recent report by Sriraksa *et al*. demonstrated that the high dose of quercetin significantly decreased the AChE activity in the hippocampal homogenate in an animal model of Parkinson's disease [81]. Besides, Lee *et al*. suggested that quercetin might regulate α3,β4-nicotinic ACh receptor and show the pharmacological actions in nervous systems by this regulation as one of the mechanisms for quercetin [82]. 

### AChE Inhibitors of Animal Origin

#### Fasciculins

Nature has gifted some animals with AChE-inhibiting components. Doubtlessly, the most interesting animal-based compounds that can potently inhibit reversibly AChE at picomolar level are fasciculins, the two toxins detected in the venom of the snake *Dendroaspis angusticeps* (green mamba) [83]. 1 g of this venom was found to contain approximately 40 mg of fasciculins, 2/3 of which was fasciculin 2 (FAS-II). 

Fasciculins are members of the superfamily of three-fingered peptidic toxins from Elapidae venoms and structural analysis elucidated that fasciculin 1 (FAS-I) and FAS-II are the polypeptides consisting of 61 amino acid residues and four disulfides. The molecular weights were calculated as 6765 Da (FAS-I) and 6735 Da (FAS-II). Sequences of the two toxins were shown to differ only at one position by a replacement of Tyr with Asp/Asn. The aromatic residues, Trp286, Tyr72, and Tyr124, located at the enzyme have been shown to possess the most substantial influence on fasciculin binding, whereas Asp74, a charged residue in the vicinity of the binding site had little impact on binding by fasciculins [84]. After binding of fasciculin, elusive structural rearrangements of AChE were suggested to explain the observed residual catalytic activity of the fasciculin-enzyme complex [85]. Harel *et al*. demonstrated that the high affinity of FAS for AChE from electric eel was due to a remarkable surface complementarity, involving a large contact area and many residues either unique to FAS or rare in other three-fingered toxins [86]. In another study [87], the crystal structure of FAS-II-mouse AChE complex delineated a large contact area consistent with the low dissociation constant of the complex and only a few of the amino acid residues which make up the overall interactive site of the FAS-II provided strong interactions needed for high binding affinity and inhibition of the enzyme. In more publications, FASs were reported to bind to the gorge entrance of AChE with excellent complementarity through many polar and hydrophobic interactions and to inhibit AChE by combined allosteric and dynamical means [88-92].

Fasciculin 3 (FAS-III), another AChE inhibitor, was also isolated from the venom of *D. polylepis* (black mamba) and *D. viridis* (western green mamba), however, its full structure has not been elucidated up to date [93]. 

#### Onchidal

**Fig. (7):**
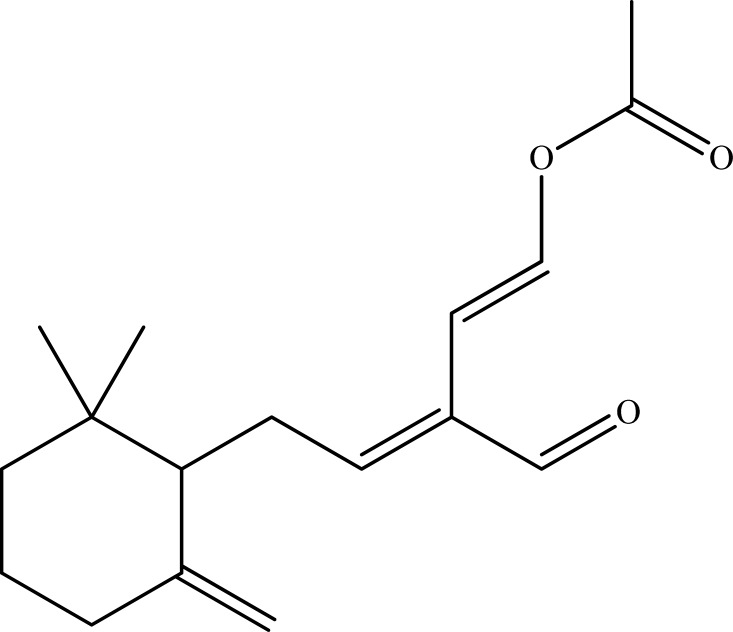
Onchidal.

Onchidal (Fig. **7**) is a natural toxin isolated from the Opisthobranch marine mollusc *Onchidella binneyi *(Onchidiacea) [94]. Considering its structural similarity to ACh, onchidal was investigated against AChE and explored to inhibit the enzyme in irreversible manner [95]. It was also stated by the authors that a potentially reactive α,β-unsaturated aldehyde group of onchidal may cause an irreversible inhibition of AChE, which result from formation of a novel covalent bond between the toxin and the enzyme and hence, this novel toxin could possibly be exploited as a new class of anticholinesterase agents.

#### Ant Venom Alkaloids

Many ant species carry various defensive toxins and repellents to survive in the nature. The ant venom alkaloids have been shown to possess diverse biological activities including AChE inhibitory effect [96-100]. For instance; 2,5-dialkylpyrrolidines and pyrroline derivative alkaloids present in the venom of the ant *Monomorium minutum* were discovered to be strong and competitive inhibitors of AChE [100]. 

#### Gilatide

Gilatide is a peptide-type of substance consisting of 9 amino acid residues, which was obtained from a kind of reptile *Heloderma suspectum*, known as “Gila monster”. It has been discovered by a neuroscientist at Thomas Jefferson University in the year of 2001 [101] and then, developed further by Axonyx Pharmaceutical Inc. (USA), which is under patent protection [102]. Therefore, not much scientific information is available for this compound at the present time.

#### Sponge Metabolites

Sponges are the primitive marine animals from Porifera with about 5000 species known across the world, although approximately 150 species of them live in fresh waters [103]. Many species have been found to contain toxic substances, probably to discourage their predators. Among them, AChE-inhibiting substances have been reported from a variety of sponges. Several 3-alkylpyridinium oligomers isolated from the Adriatic sponge *Reniera sarai* were identified as the potent inhibitors of AChE [104]. In continuation of this work, structure of the active oligomer was revealed to compose of repeating subunits of N-butyl(3-butylpyridinium) [105]. The kinetic studies on this active oligomer indicated a reversible inhibition at first, and then a slow binding and irreversible binding finally. The same research group also isolated aplysamine-4 from an unidentified verongid sponge from the Red Sea and reported that aplysamine-4 tested against insect recombinant and electric eel AChE was a non-competitive reversible inhibitor of this enzyme [106]. 

**Fig. (8):**
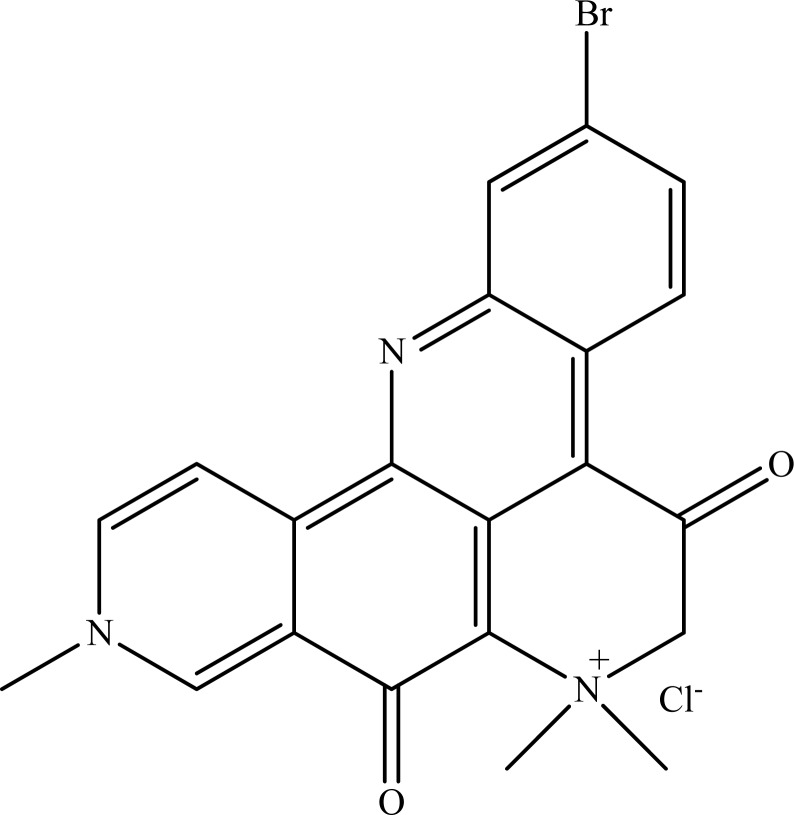
Petrosamine.

The sponge *Corticium* sp. collected from Thailand waters was found to contain a new stigmastane-type steroidal alkaloid, named as 4-acetoxy-plakinamine B with a high AChE inhibitory effect (IC_50_ = 3.75±1.69 µM) [107]. The compound showed a mixed-competitive mode of inhibition. Another Thai sponge, *Petrosia* sp., was found to contain petrosamine (Fig. **8**), a pyridoacridine alkaloid, with strong AChE inhibitory activity approximately six times higher than that of the reference galanthamine and quaternary ammonium group of petrosamine suggested to display a critical role in this inhibition [108]. 

**Fig. (9):**
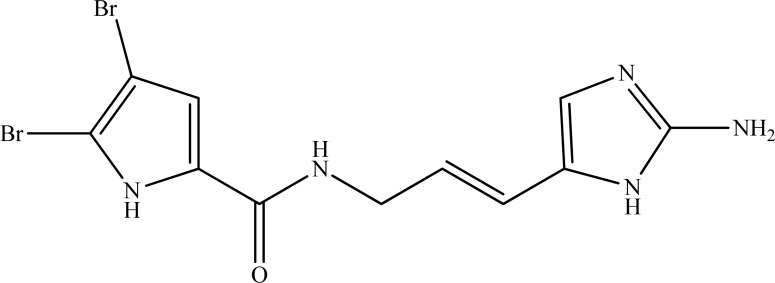
Oroidin.

In our recent report on screening of Turkish marine sponges collected from the Aegean and Mediterranean Sea, we identified oroidin (Fig. **9**) isolated from *Agelas oroides* with a moderate level of AChE inhibition (55.21±2.94% at 200 µg/mL) [109]. 

#### Arisugacins

**Fig. (10):**
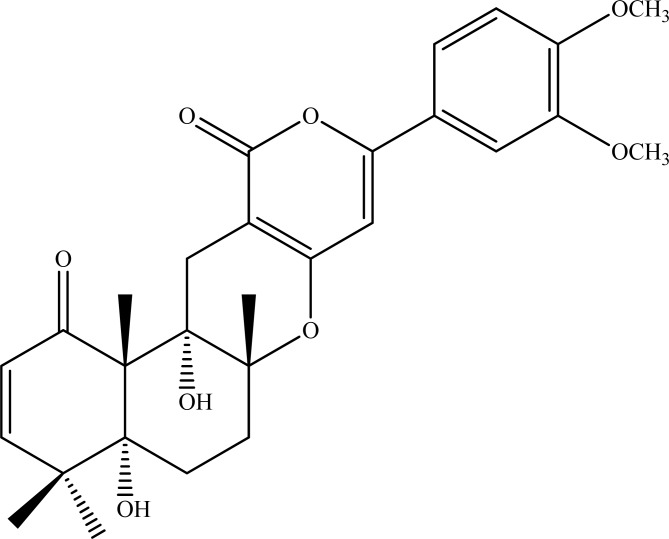
Arisugacin A.

Arisugacins A (Fig. **10**) and B are the meroterpenoid-type of compounds isolated from the microfungi *Penicillium* sp. FO-4259, which were revealed to have strong AChE inhibition [110]. 

**Fig. (11):**
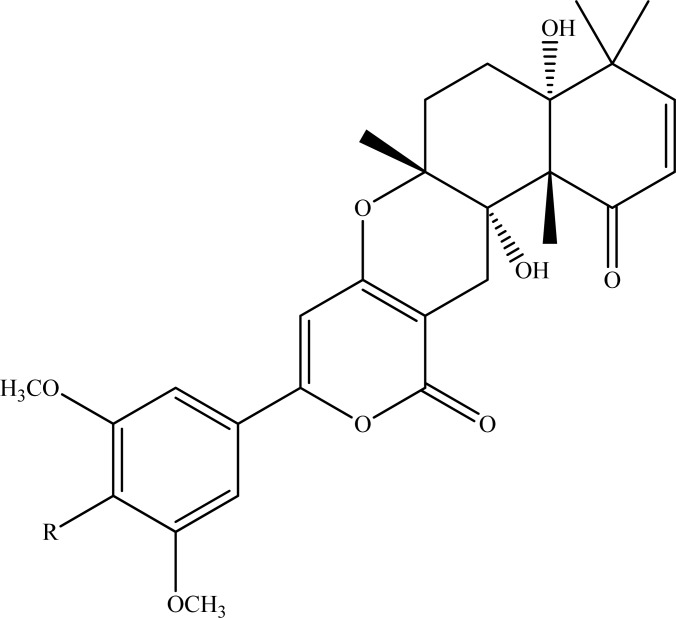
Territrems B (R=OCH3) and C (R=OH).

In addition to arisugacins, territrems B and C (Fig. **11**) and cyclopenin isolated from the same microfungus strain were also tested in the same manner. All isolated compounds exerted potent inhibitory activities against AChE with IC_50_ values in range of 1.0 approximately 25.8 nM. A recent computation docking study suggested that (+)-arisugacin A is a dual binding site covalent inhibitor of AchE with a unique mode of action [111].

## CONCLUSION

Nature has great potential to provide bioactive compounds desirable for human health. Discovery of novel inhibitors of AChE is important in developing new drug candidates for the treatment of AD, MG, and glaucoma as well new insecticidal drugs. As emphasized in this review, plants, animals, and microorganisms absolutely have potential to produce natural compounds showing definite AChE-inhibiting features and these sources need to proceed to more advance research. Therefore, AChE inhibitors of natural origin may contribute to the design of new pharmaceuticals and bring in information which might assist to understanding of these processes acknowledging and their potential role in leading new treatments.

## ABBREVIATIONS

ACh: = Acetylcholine
AChE: = Acetylcholinesterase
AD: = Alzheimer’s disease
FAS: = Fasciculin
FDA: = U.S. Food and Drug Administration
HupA: = Huperzine A
IC: = Inhibitory concentration
MG: = Myastenia gravis
PAS: = Peripheric anionic site
*Tc*AChE: = *Torpedo californica* (electric eel) acetylcholinesterase
